# TIMELESS is a key gene mediating thrombogenesis in COVID-19 and antiphospholipid syndrome

**DOI:** 10.1038/s41598-022-21694-3

**Published:** 2022-10-14

**Authors:** Wenjing Zhang, Longjiang Di, Zhongshuang Liu, Qi sun, Yan Wu, Nuan Wang, Meili Jin, Lingling Gao, Mengyu Zhang

**Affiliations:** 1 Department of Laboratory Diagnosis, The Second Affiliated Hospital of Harbin Medical University, Harbin, China; 2 Department of Stomatology, Shenzhen University General Hospital and Shenzhen University Clinical Medical Academy, Shenzhen, China; 3 Department of Emergency, The First Affiliated Hospital of Heilong Jiang University of Chinese Medicine, Harbin, China; 4 Department of Ultrasound, The Second Affiliated Hospital of Harbin Medical University, Harbin, China; 5 Space Engineering University, Beijing, China

**Keywords:** Computational biology and bioinformatics, Immunology

## Abstract

Abnormal coagulation and increased risk of thrombosis are some of the symptoms associated with COVID-19 severity. Anti-phospholipid antibodies (aPLs) present in critically ill COVID-19 patients contribute to systemic thrombosis. The aim of this study was to identify key common genes to characterize genetic crosstalk between COVID-19 and antiphospholipid syndrome (APS) using bioinformatics analysis and explore novel mechanisms of immune-mediated thrombosis in critically ill COVID-19 patients. The transcriptome data of mononuclear cells from severe COVID-19 patients and APS patients were evaluated to obtain the common genes. The protein–protein interaction network and cytoHubba module analysis in Cytoscape software were used to find the associated hinge genes and hub genes. Among the common differentially expressed genes, TIMELESS depletion was identified only in patients with severe COVID-19 and not in patients with mild COVID-19, and it was validated with the GSE159678 dataset. Functional analyses using gene ontology terms and the Kyoto Encyclopedia of Genes and Genomes pathway suggested that TIMELESS might contribute to the production of antiphospholipid antibody and thrombosis in both COVID-19 and APS patients. The potential role of TIMELESS and autophagy genes in momonuclear cells were further investigated, and GSK3B was found to be associated with TIMELESS. Autophagy targeting agents have a therapeutic potential against COVID-19 and thrombogenesis in APS, which may be related to the role of autophagy genes in the modification of circadian clock proteins. Interference with TIMELESS and other genes associated with it to regulate autoantibody expression may be a potential strategy for immunotherapy against thrombogenesis in severe COVID-19 patients.

## Introduction

The highly pathogenic and infectious novel coronavirus disease 2019 (COVID-19) has created a major health crisis worldwide. As of February 2022, more than 424,822,000 patients were confirmed to be infected with the severe acute respiratory syndrome coronavirus 2 (SARS-CoV-2), and among them, more than 5,890,300 cases of death were reported (https://covid19.who.int/). Although the novel coronavirus is considered as a pneumophilic virus, severe COVID-19 is not only a viral pulmonary infection, but also causes pathological clinical manifestations in multiple organs, accompanied by major coagulation abnormalities and thromboembolic events, such as pulmonary embolism, carotid thrombosis, deep vein thrombosis, and pregnancy thrombosis^1–4^. The incidence rate of thrombotic complications in severe COVID-19 patients was reported to be 31% by Klok et al.^5^, which is much higher than the incidence of thrombotic complications in critically ill patients prior to the COVID-19 pandemic. The incidence of abnormal coagulation events in patients who died of COVID-19 was reported to have reached 71.4%^6^.

Antiphospholipid syndrome (APS) is a systemic autoimmune disease characterized by arteriovenous or venous thrombosis with elevated persistent antiphospholipid antibodies (aPLs). Combined with previous studies, we found that there were three similarities between APS and COVID-19. Firstly, patients with APS or COVID-19 showed a marked clinical heterogeneity. Patients with APS or COVID-19 developed spontaneous thrombosis of large vessels, affecting a single site, while a small percentage of patients developed rapidly progressive, life-threatening, and multi-organ micro-vessel thrombosis^1–4,7^. Secondly, APLs can be detected in the serum of patients with COVID-19, especially in critical COVID-19 patients, with a detection rate close to 47% (31/66)^8^. Thirdly, both APS and COVID-19 infection have a significant impact on the autoimmune system. Therefore, it is critical to investigate the biological pathways mediating the pathological mechanisms of APS and COVID-19. This would explain whether COVID-19 infection induced the expression of aPLs or whether aPLs were directly involved in mediating the hemostasis abnormalities observed in COVID-19 patients. Our study aimed to uncover novel mechanisms of immune-mediated thrombosis in severe COVID-19 patients, and identify potential prognostic biomarkers for this devastating disease.

**Figure 1:**
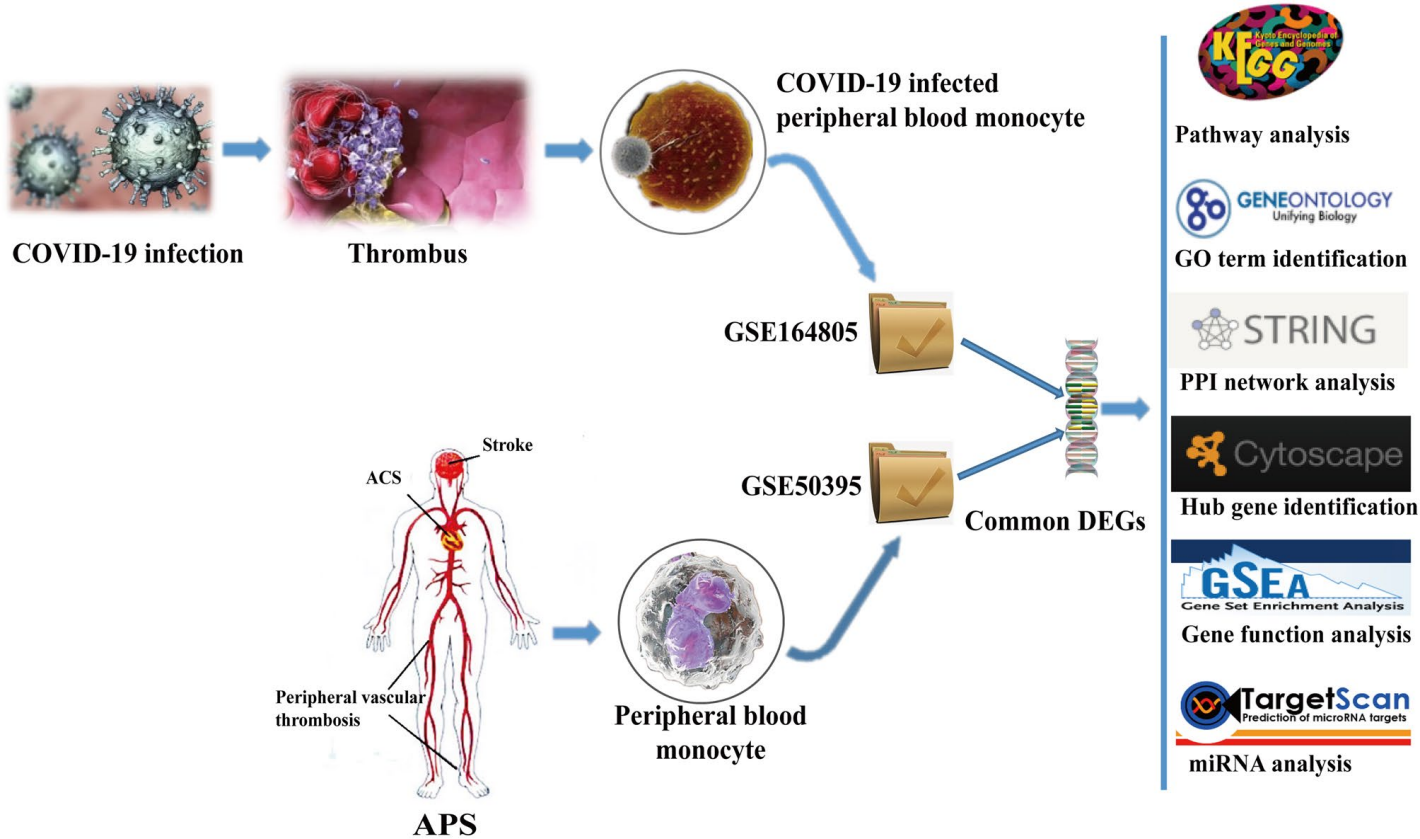
Workflow of the current investigation. Mononuclear cell samples were collected from peripheral blood of patients with severe COVID-19 included in the GSE164805 dataset. The GSE50395 dataset contains the peripheral blood monocyte samples from APS patients. Common DEGs were identified from both the datasets using the GEO2R tool on the website.

Activation of monocytes and the presence of aPLs in APS patients has a synergistic effect on thrombosis. In this study, two databases were selected for analysis. GSE164805 dataset was selected for analyzing mRNA expression of mononuclear cells from SARS-CoV-2 infected patients, and GSE50395 dataset was selected for analyzing monocyte mRNA expression from APS patients. GSE159678 dataset was used as a validation dataset to verify the expression of target mRNA in patients with severe COVID-19. All the three datasets were obtained from the Gene Expression Omnibus (GEO) Database. The initial work was to identify the differentially expressed genes (DEGs) from GSE164805 and GSE50395 datasets, and then to find the common DEGs between COVID-19 and APS patients. On the basis of the commonly expressed genes, gene aggregation analysis and pathway analysis were further carried out to understand the biological processes underlying the expressed genomes. It is crucial to identify the hub genes from the common DEGs. Thus, we developed protein–protein interaction (PPI) networks to identify the key hub genes. Core genes identified from the hub genes were searched for their targeted miRNAs. Autophagy related genes in the monocytes were screened, and the association between core genes and autophagy was identified by PPI networks. The workflow of this analysis is shown in Fig. 1.

## Methods

### Databases

The GSE164805 dataset describes the transcriptomic data from human mononuclear cells in COVID-19 patients, and the GSE50395 dataset describes the transcriptomic data from human monocytes in APS patients. Demographic and clinical characteristics of all population in both studies are summarized in Supplementary Table 1. Mononuclear cells or monocytes from patients and healthy donors with matched distribution of age and gender were chosen and subjected to microarray analysis. Both the datasets were obtained from the GEO database^9^. The COVID-19 (GSE164805) dataset was provided by Zhang et al.^10^. The APS dataset (GSE50395) was provided by Perez-Sanchez et al.^11^. The COVID-19 dataset (GSE164805) included mRNA expression data of mononuclear cells from 5 samples for each of the following patients: healthy individuals; patients with mild COVID-19 symptoms; and patients with severe COVID-19 symptoms. The criteria for cut-off for GSE164805 was obtained using adjusted *P*-value < 0.01 and log2-fold change > 1.0. The APS dataset (GSE50395) contained mRNA expression data of monocytes from 3 samples for each of the following patient groups: healthy individuals; and patients with APS. The cut-off criteria for GSE50395 dataset was obtained using *P*-value < 0.01 and log2-fold change > 1.0. The GSE159678 dataset was used to validate the expression of target mRNA in monocytes from severe COVID-19 patients.

### Analysis of the differentially expressed genes between COVID-19 and APS samples by GEO2R

GEO2R (https://www.ncbi.nlm.nih.gov/geo/geo2r/) is an interactive online tool that was used to identify DEGs between the mononuclear cell samples from COVID-19 patients and healthy individuals, and the DEGs between mononuclear cell samples from healthy individuals and APS patients. Fold-change (FC) threshold was set to 2. COVID-19 adj. *P* < 0.01 and APS *P* < 0.05 were considered to be statistically significant. The differentially expressed genes were presented in the form of volcano map drawn by limma rapid differential analysis tool on the SangerBox web (http://sangerbox.com/). The common genes between COVID-19 and APS patients were analyzed by Venn diagram (http://bioinformatics.psb.ugent.be/webtools/Venn/).

### Searching the gene ontology and gene pathway with GSEA analysis

Gene annotation uses the term gene ontology (GO), which can be divided into three categories including biological processes, molecular functions, and cellular components^12^. GO analysis helps to understand the gene-related molecular activities, cellular roles, and cellular localization of the gene of interest, separately. The Kyoto Encyclopedia of Genes and Genomes (KEGG) pathway (www.kegg.jp/feedback/copyright.html) is commonly used to understand metabolic pathways and contains important gene annotations^13^. The GO term and KEGG pathway were obtained through the network platform DAVID (https://david.ncifcrf.gov/tools.jsp) for the common DEGs that were identified in the previous step. Enrichment analysis bubble diagram were drawn using the Sangerbox tools, a free online platform for data analysis (http://sangerbox.com/Tool).

### Construction of protein–protein interaction (PPI) networks and key pathway analysis

Constructing the PPI networks is a prerequisite for the systematic study of cell biology, which is mainly applied to protein–protein and protein-DNA interactions. The STRING (https://string-db.org/) retrieval tool was used to generate functional gene interaction networks and predict information regarding gene interaction. Interaction relationships were defined through interaction attachment information, and by using the confidence ratings generated by Web tools. In order to obtain a better visual expression of the PPI networks and to identify the hinge genes, we conducted a secondary analysis on part of the obtained PPI using the Cytoscape software (http://apps.cytoscape).

### Identification of the hub genes

The hub genes were analyzed using cytoHubba (http://apps.cytoscape. org/apps/cytohubba), which is a plugin of Cytoscape software. Based on the degree topology algorithm of Cytohubba, the top five genes in the PPI networks were calculated as the hub genes. Hub genes create concentrated regions that can be detected as an important module of the PPI networks.

### Gene set enrichment analysis (GSEA) of TIMELESS

GSEA (http://sangerbox.com/Tool) was used to predict the functional effect of the single gene. The matrix data was processed by GSEA simple analysis. The most significant pathways were screened by *P* < 0.05. The visualized pictures were made by using the SangerBox tool of GSEA.

### Statistical analysis

The statistical tests for the analyzed data from the patients were plotted using Prism v.8 (GraphPad software) and the tests are described in the legends. Sample size and P values are cited in the figures and figure legends.

## Results

### Identification of the DEGs and the common genes between severe COVID-19 and APS patients

**Figure 2:**
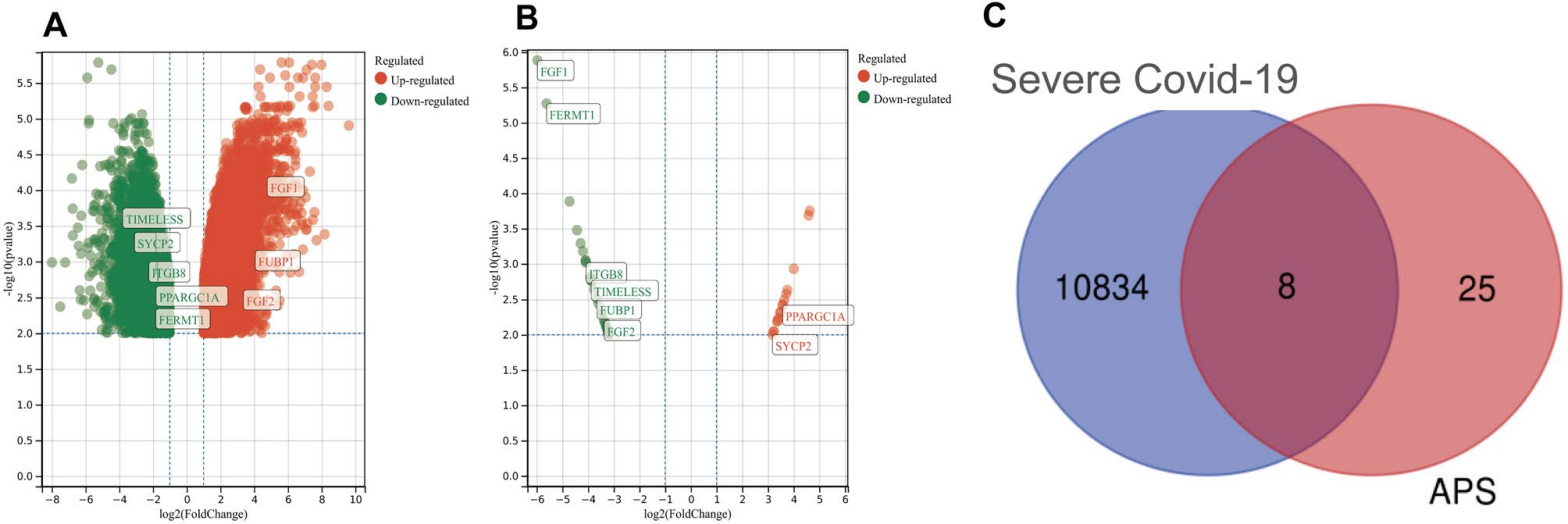
Analysis of differentially expressed genes between severe COVID-19 and APS patients. (**A**) Volcano-plot representation of the differentially expressed genes (DEGs) in severe COVID-19 patients. Green indicates that the genes were up-regulated, and red means that the genes were down-regulated. (**B**) Volcano-plot representation of the DEGs in APS patients. Y-axis represents the *P*-value (-log10 scale) and X-axis represents the fold change (log 2 scale). (**C**) Representation of the common DEGs in severe COVID-19 and APS patients through a Venn diagram. 10,842 genes were included exclusively in the severe COVID-19 group; 33 genes were included exclusively in the APS group and 8 genes were common between the severe COVID-19 and APS groups. Blue circle denotes the number of DEGs in the severe COVID-19 group and pink circle denotes the number of DEGs in the APS group.

The GSE164805 dataset was used to identify DEGs between COVID-19 patients and healthy individuals. A total of 11,056 DEGs were obtained, including 6105 up-regulated genes and 4951 down-regulated genes. A total of 53 differentially expressed genes were identified in the APS dataset GSE50395, among which 18 genes were up-regulated and 35 genes were down-regulated. The differential genes are shown in the volcano plot in Fig. 2A,B. Duplicated or invalid genes were screened (214 DEGs were excluded from the COVID-19 dataset and 20 DEGs were excluded from the APS dataset). A total of 8 common DEGs (Table 1) were identified. The Venn diagram in Fig. 2C was utilized to visually compare the common DEGs between the two datasets.

**Table 1:** Common DEGs between severe COVID-19 and APS.

Names	Total	Elements
Severe COVID-19 versus APS	8	PPARGC1A, FGF2, FUBP1, ITGB8, SYCP2, FGF1, TIMELESS, FERMT1

### GO and KEGG gene enrichment analysis

**Figure 3:**
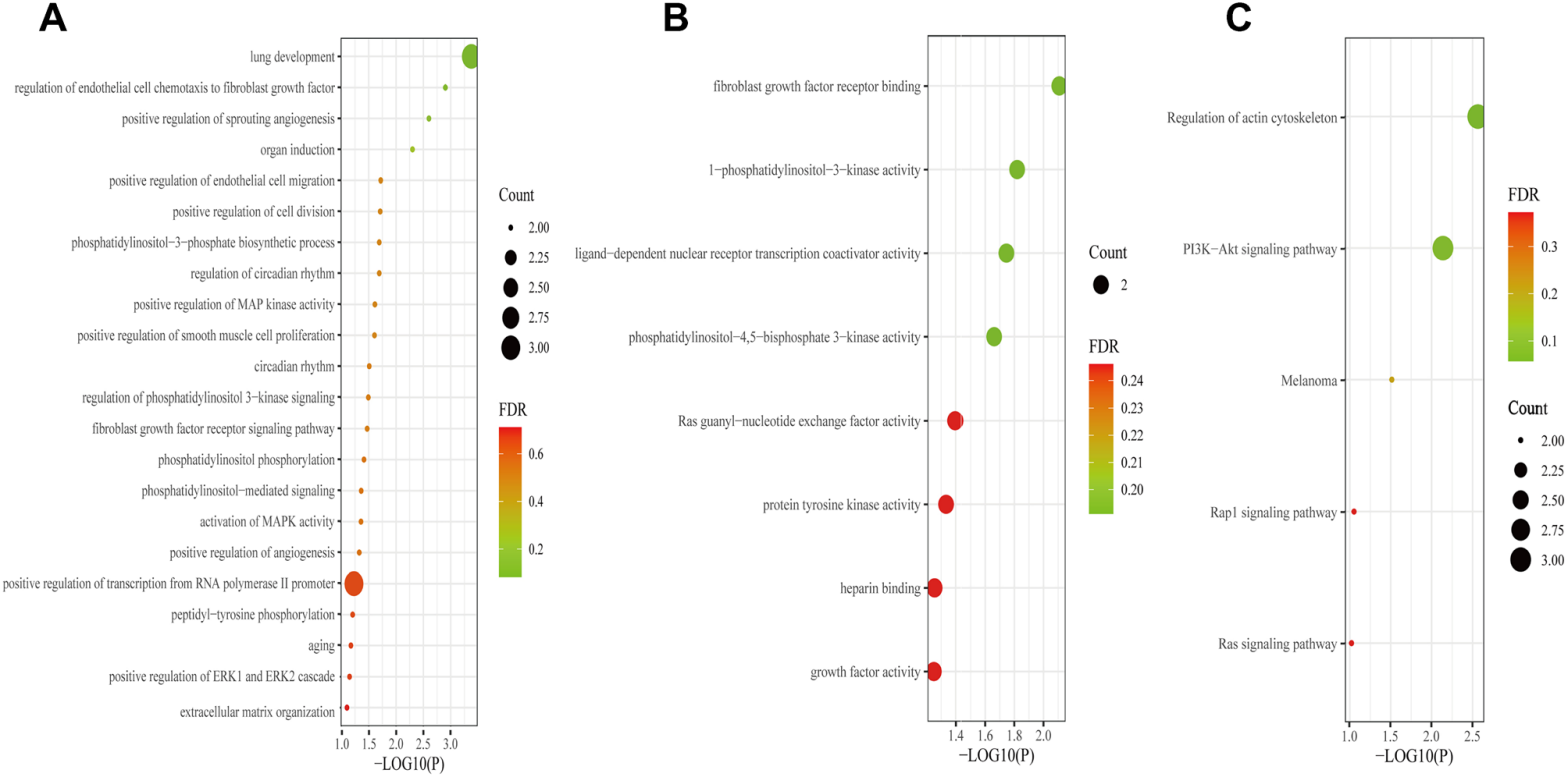
Functional characterization of the common DEGs. (**A**) Results from biological process related GO term identification derived from P-adjust and combined gene counts. (**B**) Results from molecular function related GO term identification. (**C**) Pathway analysis result identification through KEGG. X-axis represents the *P*-value (-log10 P) and Y-axis represents the term of gene function. The color of the bubble represents the FDR value, and the bubble size indicates the number of genes enriched in the same function.

David and Sangerbox enrichr web tools were used for gene set enrichment analysis. GO term and KEGG pathway of 8 (PPARGC1A, FGF2, FUBP1, ITGB8, SYCP2, FGF1, TIMELESS, FERMT1) common DEGs were analyzed in this study. The GO term was used to analyze the biological process (BP), cellular component (CC), and molecular function (MF) of the differential genes, which are presented in Table 2. The data from the GO biological process in Table 2 indicated that common DEGs were highly enhanced during lung development. In addition, we observed that the common DEGs were also highly enhanced during endothelial cell chemotaxis towards fibroblast growth factor. Cell composition studies exhibited significant involvement of nucleoplasm in the common DEGs. Molecular function subsection data indicated that fibroblast growth factor receptor was involved in the common DEGs. Actin cytoskeleton regulation pathway and PI3K-Akt signaling pathway interacted with most of the genes according to the KEGG pathway database, as shown in Table 3. The enrichment analysis indicated that only 6 of the 8 common DEGs (PPARGC1A, FGF2, FuBP1, ITGB8, FGF1, and TIMELESS) were functional genes. A collection of GO terms and pathways according to the comprehensive score is described in Fig. 3A–C.

**Table 2:** GO enrichment analysis of common DEGs.

Category	GO ID	GO pathway	*P*-values	Genes
GO Biological Process	GO:0030324	Lung development	4.18E−04	TIMELESS, FGF1, FGF2
	GO:2000544	Regulation of endothelial cell chemotaxis to fibroblast growth factor	0.001250149	FGF1, FGF2
	GO:1903672	Positive regulation of sprouting angiogenesis	0.002498958	FGF1, FGF2
	GO:0001759	Organ induction	0.00499256	FGF1, FGF2
	GO:0010595	Positive regulation of endothelial cell migration	0.019022296	FGF1, FGF2
	GO:0051781	Positive regulation of cell division	0.019432355	FGF1, FGF2
	GO:0036092	Phosphatidylinositol-3-phosphate biosynthetic process	0.020252031	FGF1, FGF2
	GO:0042752	Regulation of circadian rhythm	0.020252031	TIMELESS, PPARGC1A
	GO:0043406	Positive regulation of MAP kinase activity	0.024341612	FGF1, FGF2
	GO:0048661	Positive regulation of smooth muscle cell proliferation	0.024749764	FGF2, PPARGC1A
	GO:0007623	Circadian rhythm	0.030854506	TIMELESS, PPARGC1A
	GO:0014066	Regulation of phosphatidylinositol 3-kinase signaling	0.032071516	FGF1, FGF2
	GO:0008543	Fibroblast growth factor receptor signaling pathway	0.033692157	FGF1, FGF2
	GO:0046854	Phosphatidylinositol phosphorylation	0.038540135	FGF1, FGF2
	GO:0048015	Phosphatidylinositol-mediated signaling	0.04336725	FGF1, FGF2
	GO:0000187	Activation of MAPK activity	0.043768571	FGF1, FGF2
	GO:0045766	Positive regulation of angiogenesis	0.046973941	FGF1, FGF2
	GO:0045944	Positive regulation of transcription from RNA polymerase II promoter	0.058847464	FGF1, FGF2, PPARGC1A
	GO:0018108	Peptidyl-tyrosine phosphorylation	0.062073994	FGF1, FGF2
	GO:0007568	aging	0.066799619	FGF2, PPARGC1A
	GO:0070374	Positive regulation of ERK1 and ERK2 cascade	0.070722036	FGF1, FGF2
	GO:0030198	Extracellular matrix organization	0.078913137	ITGB8, FGF2
GO Cellular Component	GO:0005654	Nucleoplasm	0.077178849	FUBP1, TIMELESS, FGF1, PPARGC1A
GO Molecula Function	GO:0005104	Fibroblast growth factor receptor binding	0.00779516	FGF1, FGF2
	GO:0016303	1-phosphatidylinositol-3-kinase activity	0.015188693	FGF1, FGF2
	GO:0030374	Ligand-dependent nuclear receptor transcription coactivator activity	0.017993173	FGF2, PPARGC1A
	GO:0046934	Phosphatidylinositol-4,5-bisphosphate 3-kinase activity	0.021838464	FGF1, FGF2
	GO:0005088	Ras guanyl-nucleotide exchange factor activity	0.040190367	FGF1, FGF2
	GO:0004713	Protein tyrosine kinase activity	0.046357431	FGF1, FGF2
	GO:0008201	Heparin binding	0.055546091	FGF1, FGF2
	GO:0008083	Growth factor activity	0.056223787	FGF1, FGF2

**Table 3:** KEGG enrichment analysis of common DEGs.

Databases	Pathways	*P*-value	Genes
KEGG pathway	Regulation of actin cytoskeleton	0.002726797	ITGB8, FGF1, FGF2
	PI3K-Akt signaling pathway	0.007274867	ITGB8, FGF1, FGF2
	Melanoma	0.030649727	FGF1, FGF2
	Rap1 signaling pathway	0.088828225	FGF1, FGF2
	Ras signaling pathway	0.095371624	FGF1, FGF2

### PPI networks analysis and hub gene identification

**Figure 4:**
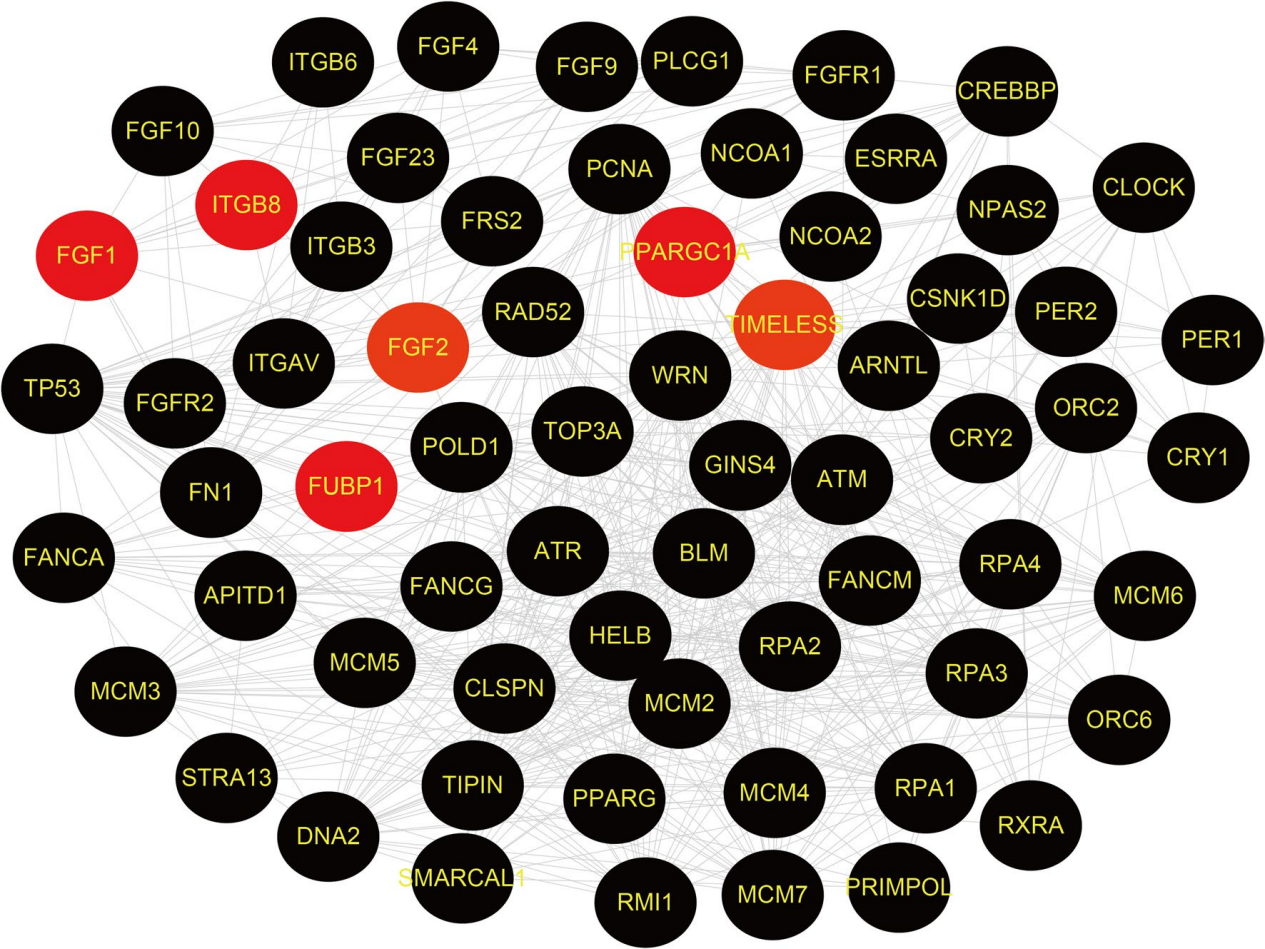
Protein–protein interaction (PPI) networks for identified common DEGs. Red colored nodes indicate common DEGs, which are the functional genes during enrichment. Black colored nodes represent the genes interacting with DEGs predicted through STIRNG website. The edge between the two nodes represents the interaction between the genes.

**Figure 5:**
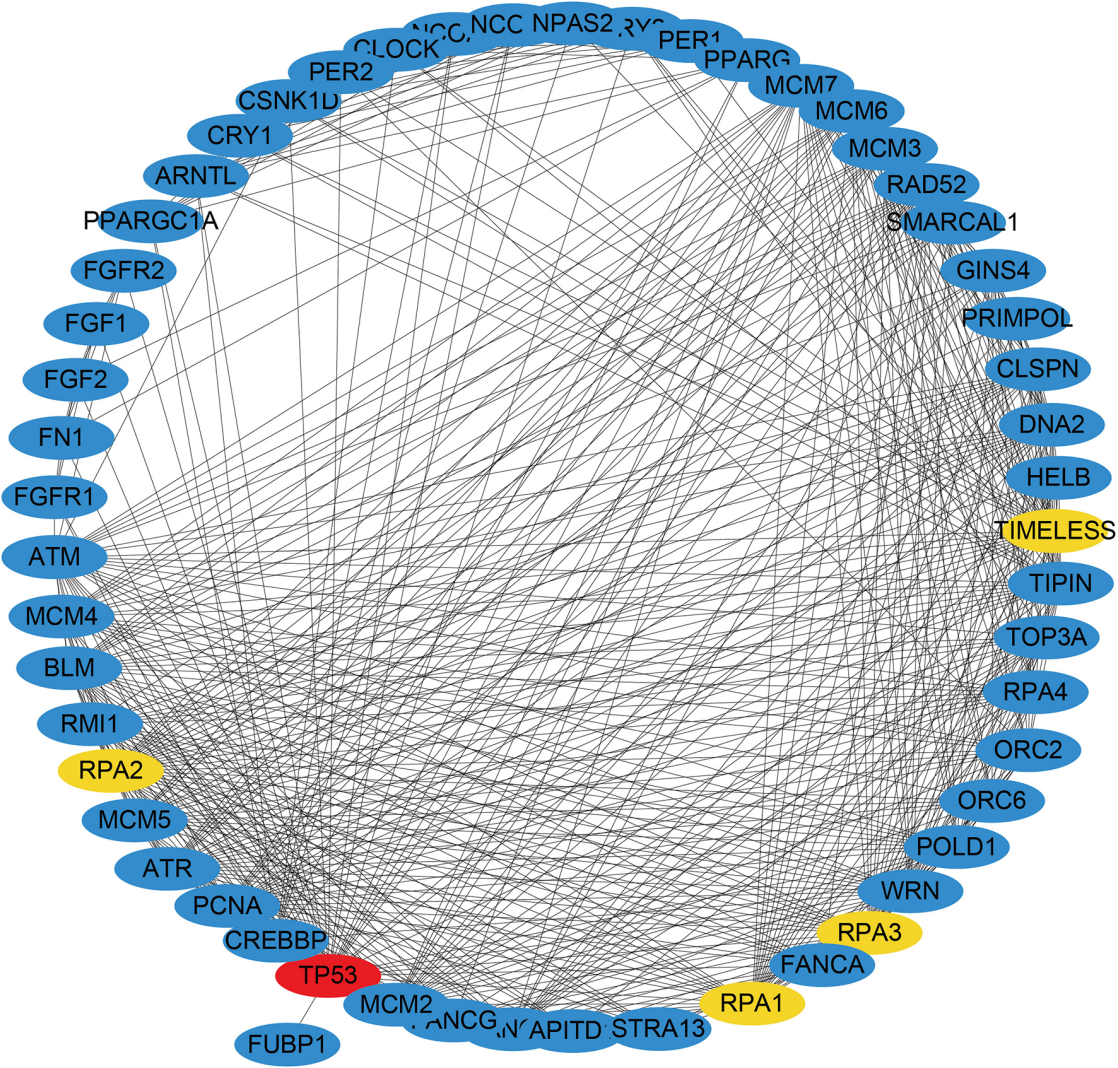
Analysis of hub genes from the PPI networks of common DEGs according to the degree value. The highlighted nodes represent the identified hub genes. Red color node represents the gene with the largest degree value in the PPI networks.

Six common DEGs were provided as input to the STRING, and the file produced from the analysis was reintroduced into the Cytoscape software for visual representation. In the PPI analysis of the screened genes, using the STRING platform, we set the confidence to 0.7. 65 nodes and 570 edges were obtained, as shown in Fig. 4. To track the hub genes from the PPI networks which is highlighted in Fig. 4, we used CytoHubba, a plugin of the Cytoscape software. The top 5 hub genes which were sorted by their degree value were TP53, TIMELESS, RPA1, RPA2 and RPA3. TIMELESS was the common DEG between COVID-19 and APS samples. Hub proteins interactions with other proteins in the PPI networks are shown in Fig. 5. The network consisted of 53 nodes and 504 edges. Topological analysis of the hub genes (TP53, TIMELESS, RPA1, RPA2 and RPA3) was performed using CytoHubba. The results from the topology analysis are presented in Table 4.

**Table 4:** Topological diagram of the top five hub genes.

Hub gene	Stress	Betweenness centrality	Closeness centrality	ClusteringCoefficient	Degree	EcCentricity
TP53	13,350	1225.77151	49.33333	0.38487	35	0.33333
TIMELESS	1668	272.51922	46.28333	0.51693	34	0.2
PRA2	1494	80.72283	46.91667	0.6631	34	0.25
PRA1	1494	80.72283	46.91667	0.6631	34	0.25
PRA3	1494	80.72283	46.91667	0.6631	34	0.25

### Comparison of TIMELESS expression in patients with mild and severe COVID-19 infection

**Figure 6:**
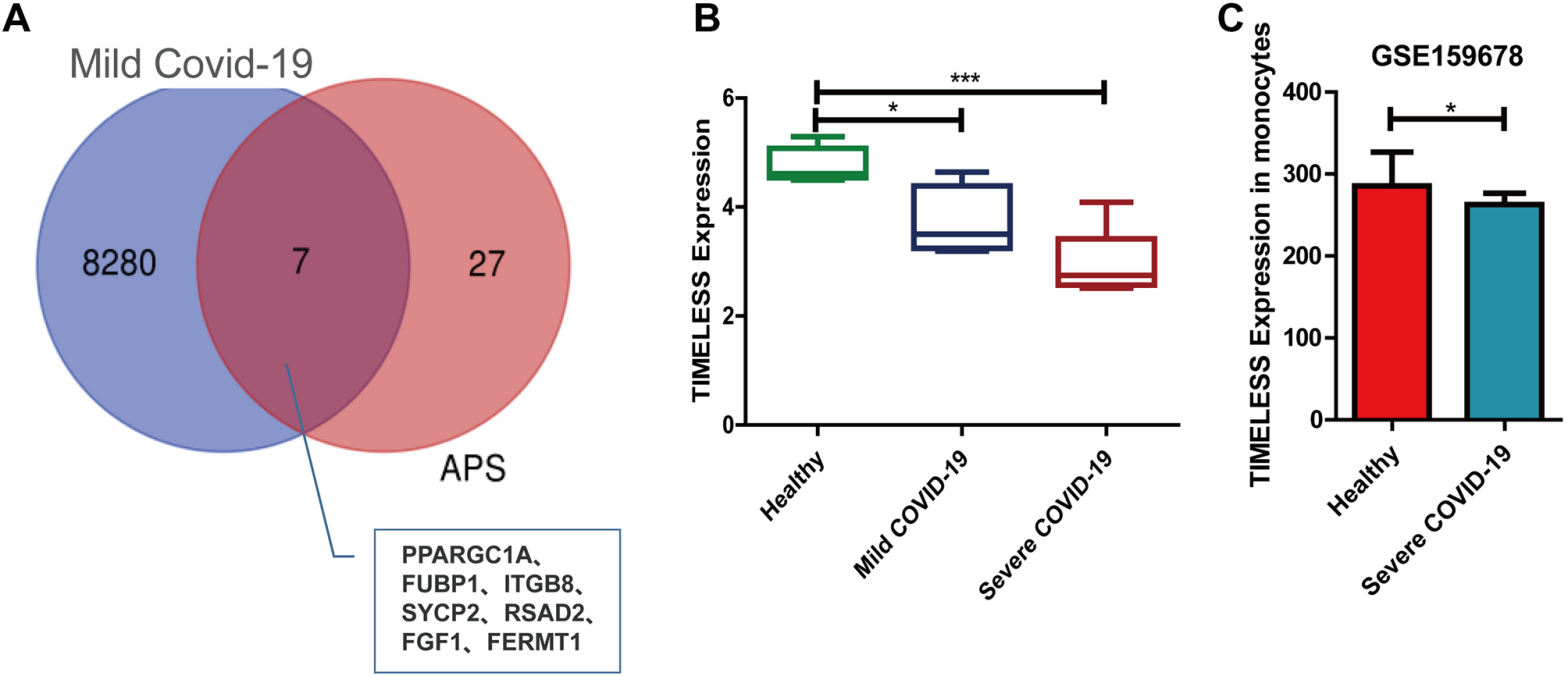
Analysis of TIMELESS gene in mild COVID-19 patients. (**A**) Venn diagram depicting the common DEGs between COVID-19 and APS patients. 8287 genes were included exclusively in the mild COVID-19 group, 33 genes were included exclusively in the APS group and 7 genes were common between the mild COVID-19 and APS groups. Blue circle denotes the number of DEGs in the mild COVID-19 group and pink circle denotes the number of DEGs in the APS group. (**B**) Expression of the TIMELESS gene in healthy, mild and severe COVID-19 patients. *P < 0.01, ***P < 0.001. (**C**) Validation of the expression of TIMELESS in monocytes from severe COVID-19 patients from the GSE159678 dataset. *P < 0.05.

Seven common DEGs (PPARGC1A, FUBP1, ITGB8, SYCP2, RSAD2, FGF1, and FERMT1) were identified from the GSE164805 dataset of patients with mild COVID-19, and the GSE50395 dataset from APS patients (Fig. 6A). TIMELESS was not found in these common DEGs. Mononuclear cells from severe COVID-19 patients were found to express lower levels of TIMELESS as compared to the patients with mild COVID-19 symptoms or healthy individuals (Fig. 6B). GSE159678 dataset validated the decrease in the expression of TIMELESS in monocytes from severe COVID-19 patients and healthy individuals (Fig. 6C).

### Correlation analysis of TIMELESS gene expression and anti-phospholipid antibodies

**Figure 7:**
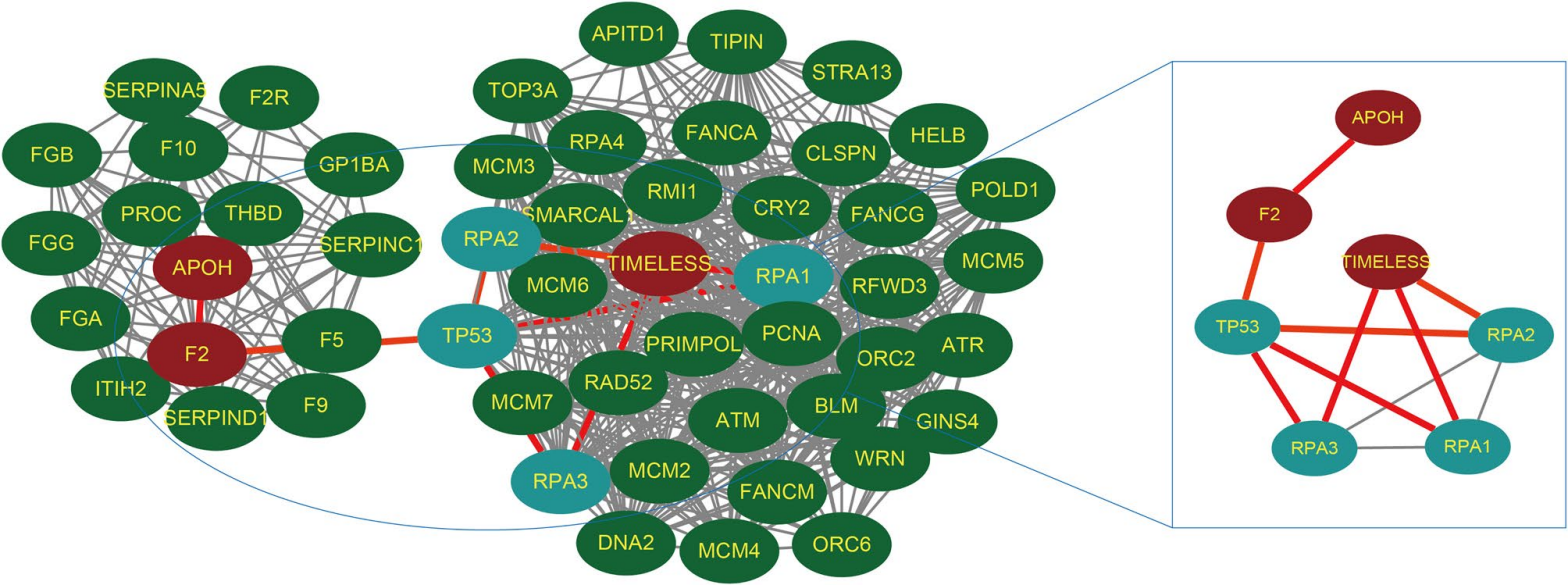
Protein–protein interaction (PPI) networks for aPLs and TIMELESS. Nodes extracted from the PPI networks are the associated nodes interacting with TIMELESS and aPLs. Red colored nodes include the target antigens of antiphospholipid antibodies: APOH (gene of β_2_GPI) and F2 (gene of lupus anticoagulant and prothrombin).

To further explore the relationship between TIMELESS and anti-phospholipid antibodies, we input TIMELESS, ApoH (gene encoding β2GPI), F2 (gene encoding lupus anticoagulant and prothrombin), and CRLS1 (gene encoding cardiolipin) as the key words into the STRING website for PPI interaction analysis. Genes encoding antiphospholipid antibodies were derived from the “Gene” module of NCBI. More protein network information was added through the "more" module. Finally, a PPI networks diagram containing 53 nodes and 510 edges was obtained. ApoH and F2, but not CRLS1, showed an association with TIMELESS expression, which was dependent on the role of the hub genes (TP53, RPA1, RPA2 and RPA3) (Fig. 7). TP53, an intermediate node associate of the antiphospholipid antibodies and TIMELESS, had a combined score of 0.67 with F2 but was not directly related to the TIMELESS. RPA3 may be more important in the relationship between antiphospholipid antibodies and TIMELESS. The combined score of RPA3 and TIMELESS was 0.999 and the co-expression score was 0.084. RPA3 also had a high combined score with the intermediate molecule TP53 (0.958).

### Functional enrichment of TIMELESS

**Figure 8:**
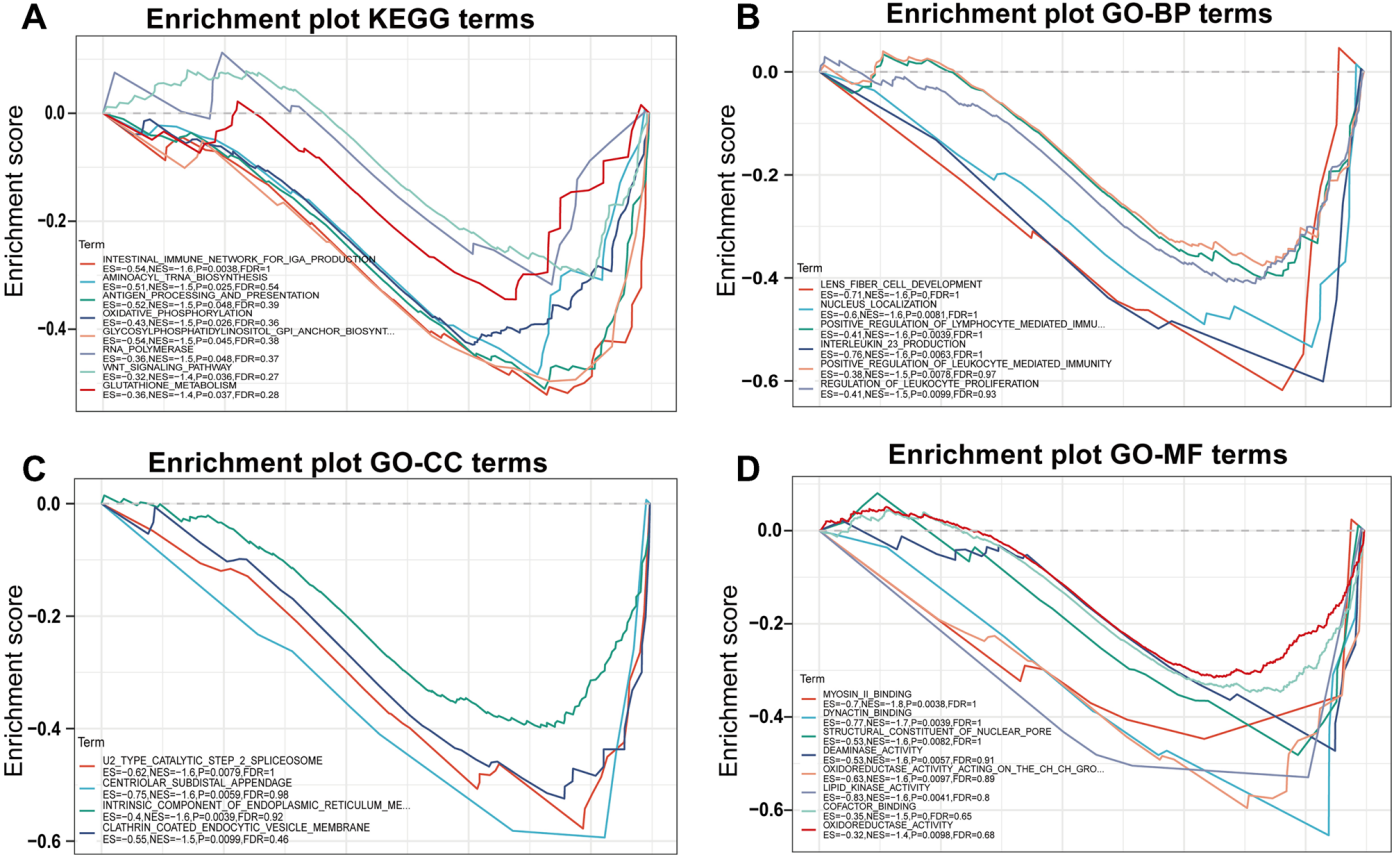
Gene set enrichment analysis (GSEA) of the TIMELESS gene. (**A**) KEGG pathway terms. (**B**) GO term of biological process. (**C**) GO term of cell component. (**D**) GO term of molecular function. The color of the line represents the term for the gene function, and Y-axis represents the enrichment score.

GSEA pathway enrichment analysis of TIMELESS gene was implemented in the sangarbox website. The up-regulated genes in the KEGG Pathway and GO term maps were plotted according to the enrichment score (ES). KEGG pathway data indicated that TIMELESS was involved in intestinal immune network for IgA production, aminoacyl t-RNA biosynthesis and antigen processing and presentation (Fig. 8A). GO term showed that TIMELESS was involved in lens fiber cell development, U2 type catalytic step 2 spliceosome and myosin II binding (Fig. 8B–D).

### Correlation analysis of TIMELESS gene and thrombogenesis

**Figure 9:**
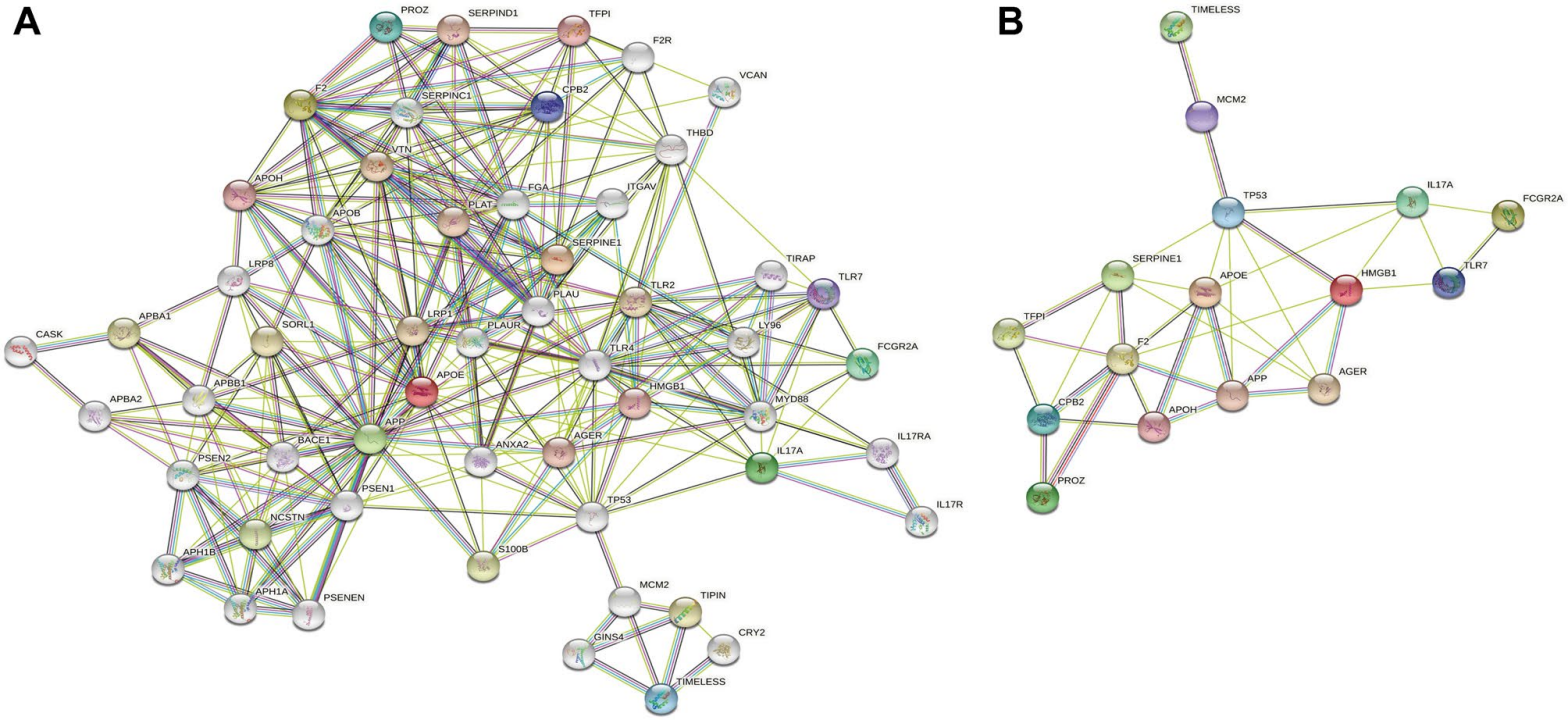
Protein–protein interaction (PPI) networks for TIMELESS and thrombogenesis related factors of APS. (**A**) The analyzed networks contained 13 candidate factors, which were obtained from the “gene” module of NCBI website. The PPI networks holds 54 nodes and 299 edges. (**B**) Simplified PPI networks from Figure A.

In order to further explore the role of TIMELESS in thrombogenesis, we identified 13 candidate genes responsible for thrombosis by searching "thrombosis AND homo AND APS" in the "Gene" module of the NCBI website (Supplementary Table 2). We found the target antigens of aPLs (APOH and F2) among these candidate genes. PPI analysis of all thrombogenesis-related genes and TIMELESS was conducted as shown in Fig. 9A. TP53 remained the common node that interacted with TIMELESS and the candidate genes (Fig. 9B).

### Correlation analysis of TIMELESS and autophagy regulatory genes

**Figure 10:**
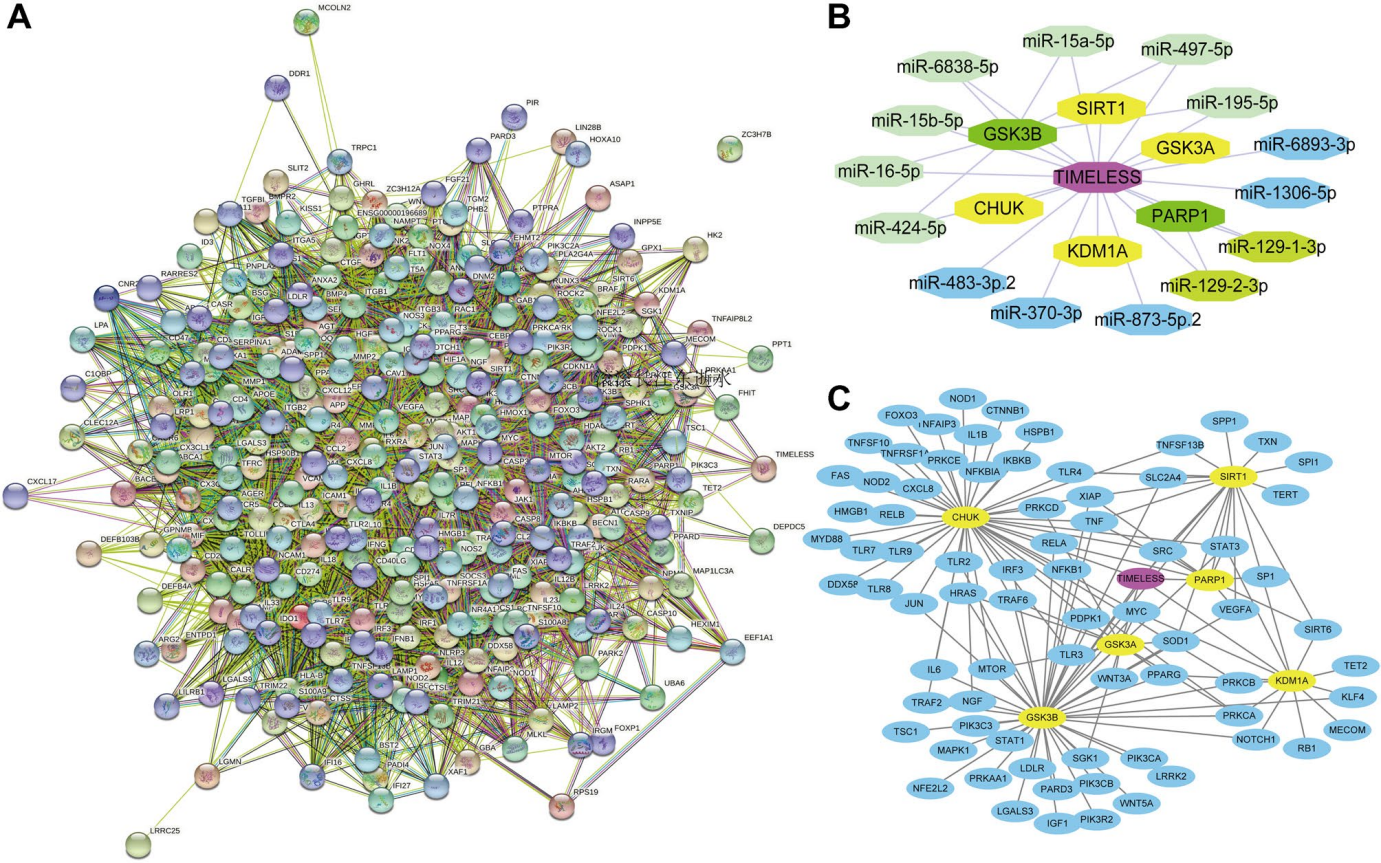
Analysis of the correlation of TIMELESS, autophagy genes and miRNA. (**A**) Protein–protein interaction (PPI) networks for TIMELESS and autophagy related genes. 319 candidate genes from monocytes which were responsible for autophagy were obtained from the “gene” module of NCBI website. (**B**) Identification of the target miRNA of TIMELESS and autophagy genes. 14 conserved miRNAs of TIMELESS were obtained from Targetscan. Blue colored nodes represent miRNAs that only interact with TIMELESS. Yellow colored nodes represent autophagy genes that only interact with TIMELESS. Green colored nodes represent the interacting autophagy genes and miRNAs. (**C**) PPI networks for TIMELESS associated genes and autophagy genes. Blue colored nodes are the genes derived from Figure A, which are directly associated with SIRT1, GSK3A, GSK3B, PARP1, CHUK and KDM1A.

During the investigation of the role of TIMELESS in APS and COVID-19, we found that several proteins involved in the regulation of autophagy were also present in the PPI networks. We obtained 319 candidate genes responsible for autophagy through searching "autophagy AND homo AND monocyte" in the "Gene" module of the NCBI website (Supplementary Table 3). PPI analysis of all autophagy-related genes and TIMELESS was conducted as shown in Fig. 10A. 6 autophagy genes (CHUK, GSK3A, GSK3B, KDM1A, PARP1 and SIRT1) were directly related to TIMELESS with a confidence greater than 0.776 in the PPI networks. We also used the Targetscan website (https://www.targetscan.org/) to find the target microRNAs (miR15a-5p, miR-424-5p, miR-6893-3p, miR195-5p, miR-16-5p, miR370-3p, miR15b-5p, miR-497-5p, miR-129-1-3p, miR-6838-5p, miR-1306-5p, miR-129-2-3p, miR-873-5p.2, and miR-483-3p.2) of TIMELESS. All the target miRNAs were compared with the autophagy genes associated with TIMELESS, which showed that GSK3B was regulated by miR15a-5p, miR-424-5p, miR-6838-5p, miR-16-5p, miR497-5p, miR15b-5p and miR-195-5p respectively; PARA1 was regulated by miR129-1-3p, and miR129-2-3P. According to the PPI networks analysis, TIMELESS also had a higher combined score with PARP1 and GSK3B (Supplementary Table 4). All the node associations were plotted using the Cytoscape software in Fig. 10B. Extended analysis was further performed for autophagy proteins, which were directly related to TIMELESS. Autophagy proteins associated with GSK3B included classical autophagy signaling molecules such as mTOR, PIK3, NF-KB and MAPK. The main autophagy proteins associated with PARP1 included VEGFA and TNF (Fig. 10C).

## Discussion

Since the beginning of the COVID-19 pandemic, increasing number of reports have drawn an association between COVID-19 and abnormal clotting parameters. Several cross-sectional studies reported a higher incidence of thrombosis in patients with COVID-19. Some studies attempted to explain the pathogenic mechanisms of COVID-19 related thrombosis, one of which was the appearance of aPLs. aPLs have long been considered as one of the mechanisms leading to pro-inflammatory and hypercoagulable states in APS^14^. aPLs positivity was found in 47% of the patients with severe COVID-19. However, to date, there is no clear association between APS and COVID-19^8^. The thrombo-pathologic process in APS is thought to be partly related to the inappropriate activation of monocytes^15^. Bioinformatics methodologies used in this study identified 10,842 genes and 33 DEGs. To explore the relationship between COVID-19 and APS, common DEGs between the GSE164805 and GSE50395 datasets were identified. After identification of 8 common DEGs (PPARGC1A, FGF2, FUBP1, ITGB8, SYCP2, FGF1, TIMELESS, and FERMT1), the remaining studies continued to analyze GO, KEGG pathway and the PPI regulatory networks. The change in target gene expression in monocytes was validated with the GSE159678 dataset.

Eight common DEGs were identified for the detection of GO terms. Among the biological processes, lung development, regulation of endothelial cell chemotaxis towards the fibroblast growth factor, and positive regulation of sprouting angiogenesis were identified as the top GO terms. TIMELESS and FGF are known to modulate lung morphogenesis, and their overexpression in lung cancer is associated with a low survival rate^16–18^. FGF restores the activity of telomerase and maintains the replicative ability of endothelial cells, and promotes neo-vascularization^19^. GO term for the cellular component was nucleoplasm. Top GO terms according to the molecular function were fibroblast growth factor receptor binding 1-phosphatidylinositol-3-kinase activity and ligand-dependent nuclear receptor transcription coactivator activity. 1-phosphatidylinositol-3-kinase is involved in the regulation of monocyte apoptosis and autophagy^20,21^.

PPI networks analysis was performed on PPARGC1A, FGF2, FUBP1, ITGB8, SYCP2, FGF1, TIMELESS and FERMT1 genes, as these genes were the common DEGs. According to the PPI networks, TP53, TIMELESS, RPA1, RPA2 and RPA3 were identified as the hub genes due to their high degree of interaction. TIMELESS is a gene that edits highly conserved proteins and regulates several cellular functions such as cell survival after damage or stress; increase in DNA polymerase epsilon activity; maintenance of telomere length; and epithelial cell morphogenesis (https://www.ncbi.nlm.nih.gov/gene/8914). We found that TIMELESS was the only common DEG presented in the hub genes, suggesting an important role of TIMELESS in the pathogenesis of both COVID-19 and APS.

Xiao et al.^8^ found that the positive rate of aPLs was 47.0% (31/66) in severe COVID-19 patients, but not in patients with milder symptoms. IgA anti-β_2_GPI antibody was the most common aPLs, presented in 28.8% (19/66) of the severe COVID-19 patients. To find out whether TIMELESS was related to aPLs production, we performed a two-step analysis. Firstly, we analyzed the common DEGs between mild COVID-19 patients and APS patients. As we expected, TIMELESS was not found in common DEGs of mild COVID-19 patients. Furthermore, our analysis showed that severe COVID-19 patients had lower levels of TIMELESS gene expression as compared to patients with milder symptoms. Relying on the PPI networks analysis, we found an interaction between TIMELESS and aPLs target antigen, specifically β_2_GPI, LA and PT. Next, we conducted GO and KEGG pathway enrichment analysis on the TIMELESS gene using GSEA website. The KEGG pathway enrichment results for TIMELESS showed that the antigen processing and presentation pathway was up-regulated. Previous reports raised the possibility that Ly-6Clo monocytes played an important role in antigen presentation, which could drive T cell-independent antibody production by B cells^22^. In severe COVID-19 patients, TIMELESS depletion may be one of the contributing factors for the increased monocyte antigen presentation capacity. Infection-induced aPLs production has been widely reported^23,24^. IgA, an immunoglobulin subtype specialized for mucosal immunity, was the most common aPLs subtype. COVID-19 mainly affects the lung and intestinal mucosa; thus the preferential production of IgA subtypes may be related to the breakage of mucosal immune tolerance. In KEGG enrichment analysis, we also found a relationship between TIMELESS and the intestinal immune axis for IgA production. The presence of IgA aPLs may suggest a new subgroup of clinically relevant severe COVID-19 patients with APS. Previous studies reported aPLs as important biomarkers for thrombosis in APS patients^14,25^. Our previous study also showed that the anti-β_2_GPI antibody promoted thrombosis by binding to its target antigen β_2_GPI^26–28^. APOH and F2 were identified as thrombogenesis-related genes in APS and were again shown to interact with TIMELESS in the PPI networks analysis. Combined with the role of TIMELESS in the production of aPLs, and data from our previous studies, we speculated that TIMELESS played a potential role in regulating the thrombotic phenotype in COVID-19 patients.

Monocytes are circulating mononuclear phagocytes with fundamental ability to differentiate into dendritic cells, and autophagy is a necessary process. Autophagy also plays an important role in innate and adaptive immunity; helps to clear intracellular pathogens directly through exogenous autophagy or participates in MHC II restrictive antigen presentation^29–32^. Inhibition of autophagy suppresses the differentiation of monocytes into dendritic cells. Autophagy occurs to promote the depletion of genes associated with TIMELESS, such as CLOCK^33^. In the PPI analysis of TIMELESS, we found an association between TIMELESS, targeted miRNAs and GSK3, which was a gene screened from the autophagy related genes that could be directly associated with TIMELESS. The GSK3 system represents a “regulating valve” that modifies the core circadian clock proteins^34^. Previous studies showed that SARS-CoV-2 used the autophagy mechanism of the host cells to promote its growth and replication^35^. Treatment of SARS-CoV-infected Vero E6 cells with GSK3 inhibitors inhibited viral replication^36,37^. Inhibition or knockdown of GSK3β, a highly conserved isoform of GSK3, reduced the transformation of endothelial cells into thrombus phenotypes, which was stimulated with anti-β_2_GPI antibodies. Several autophagy targeting drugs have been used to inhibit the replication of SARS-CoV-2 virus^38,39^. Previously approved drugs that target autophagy, may also reduce the risk of thrombosis in APS patients by directly modulating the binding of aPLs/β_2_GPI complexes to phospholipids in monocytes^40^. Continuously evolution and mutation of SARS-Cov-2 necessitates the development of drugs with good safety and efficacy profiles. Therefore, it is important to use high-throughput and virtual screening tools for the rapid development of efficacious and safe drugs that could overcome the dysfunctional immune responses associated with SARS-CoV-2 infection.

## Conclusion

Several studies have explored the important regulatory roles of key genes in COVID-19 by bioinformatics analysis. To date, there are no studies investigating the transcriptomes of COVID-19 and APS patients. We attempted to identify common genetic alterations in the monocytes from COVID-19 and APS patients. TIMELESS may interact with thrombogenic factors and impact the production of aPLs by participating in monocyte differentiation. The autophagy pathway may also be involved, since it was identified from the analysis of the interactions between autophagy genes and core genes. The correlation between disease severity and immune dysfunction in COVID-19 patients should serve as a consideration during drug development and evaluation. The pathogenic mechanisms and complications associated with COVID-19 need to be further studied. As SARS-CoV-2 is a recent discovery, there were only a few available datasets to analyze its thrombosis complications. With the availability of future datasets, the research on symptomatic and preventive treatment of COVID-19 patients would become more and more important.

## Supplementary Information

Supplementary Information 1.

Supplementary Information 2.

Supplementary Information 3.

Supplementary Information 4.

## Data Availability

All data was obtained from the Gene Expression Omnibus (GEO, https://www.ncbi.nlm.nih.gov/geo/). The names of the accession numbers can be found below: GSE50395, GSE164805, and GSE159678.
